# Supplementary material for: Limited research investigating the value of MRI in predicting future cognitive morbidity in survivors of paediatric brain tumours: A systematic-review and call to action for clinical neuroimaging researchers
Source: PLoS One. 2025 Jan 30;20(1):e0314721. doi: 10.1371/journal.pone.0314721 (PMC11781722; doi:10.1371/journal.pone.0314721)
Supplement: S1 Table — Formulation of searches for the different data sources for the study, including number of results at each search point. (DOCX) [file pone.0314721.s001.docx]

**S1 Table. Tables Outlining Search Strategy.** Formulation of searches for the different data sources for the study, including number of results at each search point.

**Web of Science**

| Block 1 Child  (TOPIC) | (pediatric OR paediatric OR infant* OR child* OR adolescen* OR youth OR teen* OR young OR neonat*) |
| --- | --- |
| Block 2 Brain  (TOPIC) | (brain OR CNS OR neuro* OR intracranial OR supratentorial OR cerebell* or “posterior fossa”) |
| Block 3 Tumour  (TOPIC) | (tumo$r OR neoplasm OR cancer* OR malignan* OR pilocytic-astrocytoma OR pituitary-tumo$r OR glioma OR neuronal-gial-tumo$r OR medulloblastoma OR ependym* OR astrocytoma OR craniopharyngioma OR germ-cell-tumo$r OR glioblastoma OR meningioma OR mesenchymal-tumo$r OR hemangioma OR atypical-teratoid*rhabdoid-tumor OR AT*RT) |
| Block 4 Cognition  (TOPIC) | (neuropsych* OR cogniti* OR “late-effects” OR mental OR IQ) |
| Block 5 MRI  (TOPIC) | (magnetic resonance imaging OR MRI OR DWI OR DTI OR diffusion*imaging OR ASL OR spectroscop* OR neuroimag* OR MRS OR perfusion OR MRSI OR GRE OR gradient-echo OR DSC OR DCE) |

**Search date:** 01/07/2022

**Number of results:** 3,449

**Search date:** 27/03/2024

**Number of results:** 77 (since previous search)

*TOPIC = title, abstract, author keywords, Keyword Plus

**Scopus**

| Block 1 Child  (TITLE-ABS-KEY) | (pediatric OR paediatric OR infant* OR child* OR adolescen* OR youth OR teen* OR young OR neonat*) |
| --- | --- |
| Block 2 Brain  (TITLE-ABS-KEY) | (brain OR CNS OR neuro* OR intracranial OR supratentorial OR cerebell* or “posterior fossa”) |
| Block 3 Tumour  (TITLE-ABS-KEY) | (tumour OR tumor OR neoplasm OR cancer* OR malignan* OR pilocytic-astrocytoma OR pituitary-tumour OR pituitary-tumor OR glioma OR neuronal-gial-tumour OR neuronal-gial-tumor OR medulloblastoma OR ependym* OR astrocytoma OR craniopharyngioma OR germ-cell-tumour OR germ-cell-tumor OR glioblastoma OR meningioma OR mesenchymal-tumour OR mesenchymal-tumour OR hemangioma OR atypical-teratoid*rhabdoid-tumor OR AT*RT) |
| Block 4 Cognition  (TITLE-ABS-KEY) | (neuropsych* OR cogniti* OR “late-effects” OR mental OR IQ) |
| Block 5 MRI  (TITLE-ABS-KEY) | (magnetic resonance imaging OR MRI OR DWI OR DTI OR diffusion*imaging OR ASL OR spectroscop* OR neuroimag* OR MRS OR perfusion OR MRSI OR GRE OR gradient-echo OR DSC OR DCE) |

**Search date:** 01/07/2022

**Number of results:** 2,941

**Search date:** 27/03/2024

**Number of results:** 503 (since previous search)

*TITLE-ABS-KEY = Title, Abstract, Keywords

**PsycINFO**

| Block 1 Child  (NOFT) | (pediatric OR paediatric OR infant* OR child* OR adolescen* OR youth OR teen* OR young OR neonat*) |
| --- | --- |
| Block 2 Brain  (NOFT) | (brain OR CNS OR neuro* OR intracranial OR supratentorial OR cerebell* or “posterior fossa”) |
| Block 3 Tumour  (NOFT) | (tumo?r OR neoplasm OR cancer* OR malignan* OR pilocytic-astrocytoma OR pituitary-tumo?r OR glioma OR neuronal-gial-tumo?r OR medulloblastoma OR ependym* OR astrocytoma OR craniopharyngioma OR germ-cell-tumo?r OR glioblastoma OR meningioma OR mesenchymal-tumo?r OR hemangioma OR atypical-teratoid*rhabdoid-tumor OR AT*RT) |
| Block 4 Cognition  (NOFT) | (neuropsych* OR cogniti* OR “late-effects” OR mental OR IQ) |
| Block 5 MRI  (NOFT) | (magnetic resonance imaging OR MRI OR DWI OR DTI OR diffusion*imaging OR ASL OR spectroscop* OR neuroimag* OR MRS OR perfusion OR MRSI OR GRE OR gradient-echo OR DSC OR DCE) |

**Search date:** 01/07/2022

**Number of results:** 811

**Search date:** 27/03/2024

**Number of results:** 69 (since previous search)

*NOFT = Anywhere except full text *(didn’t have option to limit to title, abstract and keywords)*

**Embase**

((pediatric OR paediatric OR infant* OR child* OR adolescen* OR youth OR teen* OR young OR neonat*) AND (brain OR CNS OR neuro* OR intracranial OR supratentorial OR cerebell* or “posterior fossa”) AND (tumo?r OR neoplasm OR cancer* OR malignan* OR pilocytic-astrocytoma OR pituitary-tumo?r OR glioma OR neuronal-gial-tumo?r OR medulloblastoma OR ependym* OR astrocytoma OR craniopharyngioma OR germ-cell-tumo?r OR glioblastoma OR meningioma OR mesenchymal-tumo?r OR hemangioma OR atypical-teratoid*rhabdoid-tumor OR AT*RT) AND (neuropsych* OR cogniti* OR “late-effects” OR mental OR IQ) AND (magnetic resonance imaging OR MRI OR DWI OR DTI OR diffusion*imaging OR ASL OR spectroscop* OR neuroimag* OR MRS OR perfusion OR MRSI OR GRE OR gradient-echo OR DSC OR DCE)).ti,ab,kw

**Search date:** 01/07/2022

**Number of results:** 1,028

**Search date:** 27/03/2024

**Number of results:** 177 (since previous search)

*ti,ab,kw = Title, Abstract, Keywords

IMPORTANT: If you copy-paste to rerun the search, remember to remove/retype quotation marks around “posterior fossa” and “late-effects” in search engine as it recognises these as illegal characters otherwise.

**MEDLINE**

((pediatric OR paediatric OR infant* OR child* OR adolescen* OR youth OR teen* OR young OR neonat*) AND (brain OR CNS OR neuro* OR intracranial OR supratentorial OR cerebell* or “posterior fossa”) AND (tumo?r OR neoplasm OR cancer* OR malignan* OR pilocytic-astrocytoma OR pituitary-tumo?r OR glioma OR neuronal-gial-tumo?r OR medulloblastoma OR ependym* OR astrocytoma OR craniopharyngioma OR germ-cell-tumo?r OR glioblastoma OR meningioma OR mesenchymal-tumo?r OR hemangioma OR atypical-teratoid*rhabdoid-tumor OR AT*RT) AND (neuropsych* OR cogniti* OR “late-effects” OR mental OR IQ) AND (magnetic resonance imaging OR MRI OR DWI OR DTI OR diffusion*imaging OR ASL OR spectroscop* OR neuroimag* OR MRS OR perfusion OR MRSI OR GRE OR gradient-echo OR DSC OR DCE)).ti,ab,kw

**Search date:** 01/07/2022

**Number of results:** 403

**Search date:** 27/03/2024

**Number of results:** 73 (since previous search)

*ti,ab,kw = Title, Abstract, Keywords

IMPORTANT: If you copy-paste to rerun the search, remember to remove/retype quotation marks around “posterior fossa” and “late-effects” in search engine as it recognises these as illegal characters otherwise.

**OSF Preprints (grey literature)**

((pediatric OR paediatric OR infant* OR child* OR adolescen* OR youth OR teen* OR young OR neonat*) AND (brain OR CNS OR neuro* OR intracranial OR supratentorial OR cerebell* or “posterior fossa”) AND (tumo?r OR neoplasm OR cancer* OR malignan* OR pilocytic-astrocytoma OR pituitary-tumo?r OR glioma OR neuronal-gial-tumo?r OR medulloblastoma OR ependym* OR astrocytoma OR craniopharyngioma OR germ-cell-tumo?r OR glioblastoma OR meningioma OR mesenchymal-tumo?r OR hemangioma OR atypical-teratoid*rhabdoid-tumor OR AT*RT) AND (neuropsych* OR cogniti* OR “late-effects” OR mental OR IQ) AND (magnetic resonance imaging OR MRI OR DWI OR DTI OR diffusion*imaging OR ASL OR spectroscop* OR neuroimag* OR MRS OR perfusion OR MRSI OR GRE OR gradient-echo OR DSC OR DCE))

**Search date:** 03/07/2022

**Number of results:** 1
