# Supplementary material for: Limited research investigating the value of MRI in predicting future cognitive morbidity in survivors of paediatric brain tumours: A systematic-review and call to action for clinical neuroimaging researchers
Source: PLoS One. 2025 Jan 30;20(1):e0314721. doi: 10.1371/journal.pone.0314721 (PMC11781722; doi:10.1371/journal.pone.0314721)
Supplement: S1 Fig — Results from Original July 2022 searches. (DOCX) [file pone.0314721.s002.docx]

**S2 Figure. PRISMA flow diagram.** Results from Original July 2022 searches.

**Identification of studies via other methods**

**Identification of studies via databases and registers**

Records identified from:

OSF preprints (n = 1)

Literature reviews (n = 3)

Citation/reference searching (n = 4)

Records removed *before screening*:

Duplicate records removed
(n = 2,402)

Records marked as ineligible or unable to retrieve (n = 551)

Records identified from:

Web of Science (n = 3,449)

Scopus: (n = 2,941)

Embase: (n = 1,028)

PsycINFO: (n = 811)

MEDLINE: (n = 403)

**Identification**

Records screened

(n = 5,679)

Records excluded

(n = 5,517)

Reports not retrieved

(n = 0)

Reports sought for retrieval

(n = 8)

Reports sought for retrieval

(n = 162)

Reports not retrieved

(n = 9)

**Screening**

Reports excluded:

Age at diagnosis >18 (n = 52)

Neuropsych assessment precedes MRI (n = 52)

Not peer reviewed (n = 29)

No analyses of interest
(n = 10)

No standardised neuropsych assessment (n = 4)

Case study(s) (n = 1)

Non-English text (n = 1)

Reports assessed for eligibility

(n = 8)

Reports excluded:

Age at diagnosis >18 (n = 3)

MRI precedes neuropsych test (n = 3)

No analyses of interest
(n = 1)

Literature review (n = 1)

Reports assessed for eligibility

(n = 153)

Studies included in review

(n = 4)

**Included**
